# Small mammals of background areas in the vicinity of the Karabash copper smelter (Southern Urals, Russia)

**DOI:** 10.3897/BDJ.10.e76215

**Published:** 2022-01-13

**Authors:** Svetlana Mukhacheva, Yulia Davydova, Artëm Sozontov

**Affiliations:** 1 Institute of Plant and Animal Ecology (IPAE UB RAS), Ekaterinburg, Russia Institute of Plant and Animal Ecology (IPAE UB RAS) Ekaterinburg Russia

**Keywords:** data paper, occurrence records, insectivores, rodents, biological diversity, landscape-habitat diversity, environmental heterogeneity, industrial pollution

## Abstract

**Background:**

The dataset contains records of small mammals (Eulipotyphla and Rodentia) collected in the background (unpolluted) areas in the vicinity of Karabash copper smelter (Southern Urals, Russia) and the territory of the Sultanovskoye deposit of copper-pyrite ores before the start of its development. Data were collected during the snowless periods in 2007 (18 sampling plots), 2008–2010 (13 plots annually), 2011 (30 plots) and 2012–2014 (19 plots annually). The capture of animals was carried out in different types of forests (pine, birch, mixed and floodplain), sparse birch stands, reed swamps, marshy and dry meadows, border areas, a household waste dump, areas of ruderal vegetation and a temporary camp. Our study of small mammals was conducted using trap lines (snap and live traps). During the study period, 709 specimens of small mammals were caught, which belonged to five species of shrews and 13 species of rodents. The dataset may be highly useful for studying regional fauna and the distribution of species in different habitats and could also be used as reference values for environmental monitoring and conservation activities.

**New information:**

Our dataset contains new information on occurrences of small mammals. It includes the peculiarities of their habitat distribution in the background areas in the vicinity of the large copper smelter and the deposit of copper-pyrite ores before the start of its development (Chelyabinsk Oblast, Russia). All occurrence records of 18 mammal species with georeferencing have been published in GBIF.

## Introduction

Small mammals (Eulipotyphla and Rodentia) are ubiquitous, abundant, fertile, have a short life cycle and respond quickly to abiotic and biotic factors (Lidicker 1988, Krebs 1996, Lidicker 1999). Therefore, animals of these groups are traditionally used as the model objects of various ecological studies, including studies focusing on the monitoring of terrestrial ecosystems that have been affected by anthropogenic impacts (Sheffield et al. 2001).

It is well known that structural re-arrangements occur in the communities of small mammals in response to anthropogenic impacts. The magnitude and direction of these re-arrangements depend on the type, intensity and duration of such impacts, as well as on the specific characteristics of the species that make up these communities (Burel et al. 2004, Asfora and Pontes 2009, Krojerová-Prokešová et al. 2016, Volpert and Shadrina 2020, Mukhacheva and Sozontov 2021). According to popular understanding, the animal communities exhibiting the greatest diversity and abundance are those which inhabit natural areas where there is little or no human impact. As anthropogenic load increases, more and more negative changes are observed in communities; these can often be non-linear in nature (Lukyanova and Lukyanov 1998, Kataev 2005, Kozlov et al. 2005, Mukhacheva 2013, Mukhacheva 2021).

Long-term studies of biodiversity in communities of small mammals in areas affected by industrial pollution have convincingly demonstrated that the use of different methodological approaches to the analysis of animal communities in the same territories leads to fundamentally different conclusions. The data obtained when studying small mammals in one or two variants of dominant habitats indicated a significant depletion in species richness and a multiple decrease (by a factor of 10) in the total abundance as the technogenic load increased (Mukhacheva et al. 2010b, Mukhacheva 2013). In simultaneously examining a large range of habitats, it was found that the γ-diversity of the communities of background and most-polluted areas was similar and the total abundance of animals differed only by a factor of 2 (Mukhacheva et al. 2012). Based on the results obtained, it was concluded that it is necessary to take into account the heterogeneity of the environment in studies of the spatiotemporal dynamics of biodiversity of small mammal communities.

We managed to find the most suitable environmental conditions for this approach in the Southern Urals, in the Chelyabinsk Oblast. The territory of the region – located on the border of Europe and Asia – is distinguished by a wide variety of natural conditions, which determine the complexity and heterogeneity of the vegetation cover. The boundaries of several geographical landscape zones converge here: to the south is the forest zone, to the north lies the steppe zone and between them is a transitional strip of forest-steppe landscape.

The high diversity of animals in the Southern Urals is attributed to the existence of various natural conditions and a long (since the Neogene period) history of faunistic complexes being formed. Here, there is a mixture of European and Asian species, polar and desert fauna representatives and endemic and relict species. The modern fauna of the Chelyabinsk Oblast comprises 80 species of mammals, including 13 species of insectivores and 33 species of rodents. The Red Book of this region currently includes 17 species of mammals, including one species of Eulipotyphla – Russian desman (Desmana moschata, Eulipotyphla) and seven species of Rodentia: Siberian flying squirrel (Pteromys volans), garden dormouse (Eliomys quercinus), great jerboa (Allactaga major), grey hamster or migratory hamster (Cricetulus migratorius), Eversmann's hamster (Allocricetulus eversmanni), Djungarian hamster (Phodopus sungorus) and wood lemming (Myopus schisticolor) (Bolshakov 2017).

At the same time, the region is characterised by significant economic growth, as well as considerable industrial development (mainly metallurgy and mechanical engineering). In the northwest was (until 2017) a nickel-cobalt smelter ("Ufaleinickel"), in the east is the Kyshtym copper electrolytic plant, in the central area is the Karabash copper smelter and in the south are a number of large-scale ferrous metallurgy and engineering enterprises. In addition, in the northeast is the East Ural Radioactive Trace (EURT), an area which became contaminated in 1957 due to an accident at the Mayak chemical plant. As a result, for many decades, the ecological situation in the region remained one of the most tense in Russia (Ministry of Ecology of the Chelyabinsk oblast 2020). This significantly complicated the selection of reference (background) sites that had not undergone technogenic transformations. It was important to survey small mammal communities in the main habitats (forest, open, near-water), taking into account the high biotopic diversity of the studied territory.

By the beginning of our work (2007), detailed systematic studies of small mammal communities in the Chelyabinsk Oblast had not yet been carried out, with the exception of the territory of the Ilmensky Nature Reserve, which is also located in the eastern foothills of the Southern Urals in the pine-birch forest subzone (Tsetsevinsky 1975, Kiseleva 1989, Ushkov 1993, Samoilova 2005, Samoilova 2006). The modern fauna of the Reserve comprises 48 species of mammals, including six species of insectivores and 20 species of rodents (Ushkov 1993). Valuable sources of information on the habitat distribution of small mammals in this area are numerous ecological studies of mass species, such as herb field mouse (*Apodemusuralensis*), bank vole (*Myodesglareolus*), field vole (*Microtusagrestis*), as well as species requiring special methods of trapping – the northern mole vole (*Ellobiustalpinus*) or European mole (*Talpaeuropaea*), for example (Kolcheva and Olenev 1991, Olenev 1995, Evdokimov 2002, Maklakov et al. 2004, Grigorkina et al. 2008, Nesterkova 2014, Modorov 2016, Olenev and Grigorkina 2016, Orekhova and Modorov 2016, Cheprakov and Chernousova 2020, Orekhova 2020) .

In our research, we present data on the distribution of 18 species of small mammals over five districts of the Chelyabinsk Oblast (Kyshtymsky, Karabashsky, Kunashaksky, Argayashsky and Miassky) in 14 main habitats. These results may be of interest primarily for ecotoxicologists as reference communities of various habitats under conditions of minimal anthropogenic loads. Data from the area of the Sultanovskoye copper-pyrite ore deposit can be used to assess the environmental disturbances resulting from the operation of the mine. The quality of the material collection also enables the data to be used to study regional and global patterns of small mammal’s biodiversity.

## General description

### Purpose

The purpose is to describe a dataset comprising the occurrence records of small mammals (Eulipotyphla and Rodentia) in the main habitat types in the background (unpolluted) areas of the Chelyabinsk Oblast (Southern Urals, Russia). This dataset is part of our long-term research of small mammal communities inhabiting areas with different levels of industrial pollution.

## Project description

### Title

Small mammals of background areas in the vicinity of the Karabash copper smelter (Southern Urals, Russia)

### Personnel

Svetlana Mukhacheva, Yulia Davydova, Artëm Sozontov

### Study area description

**Figure 1.**
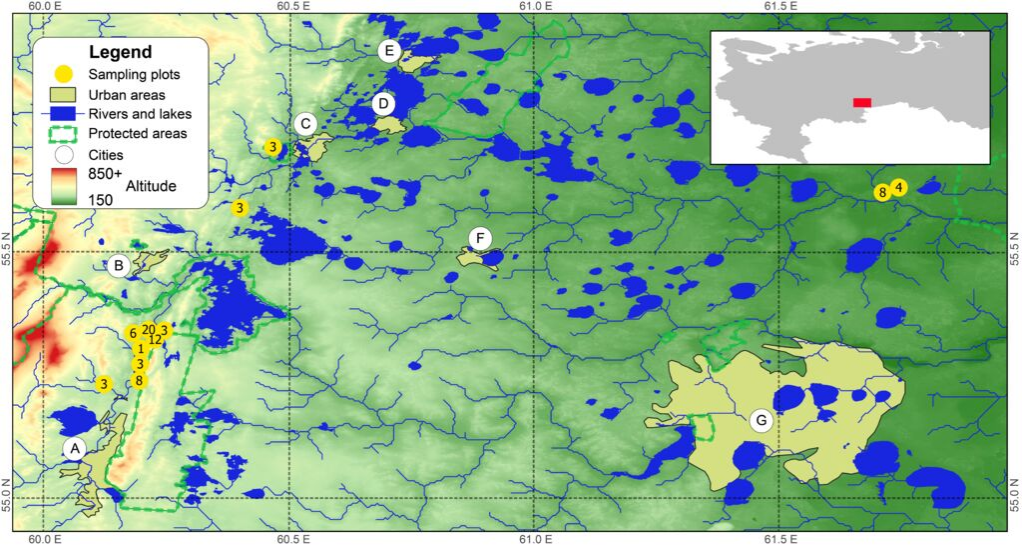
General map of the studied region and its position in the European part of Russia (see insert). Yellow polygons show urban areas of the following cities: A – Miass, B – Karabash, C – Kyshtym, D – Ozersk, E – Kasli, F – Argayashskoe, G –Chelyabinsk.

**Figure 2.**
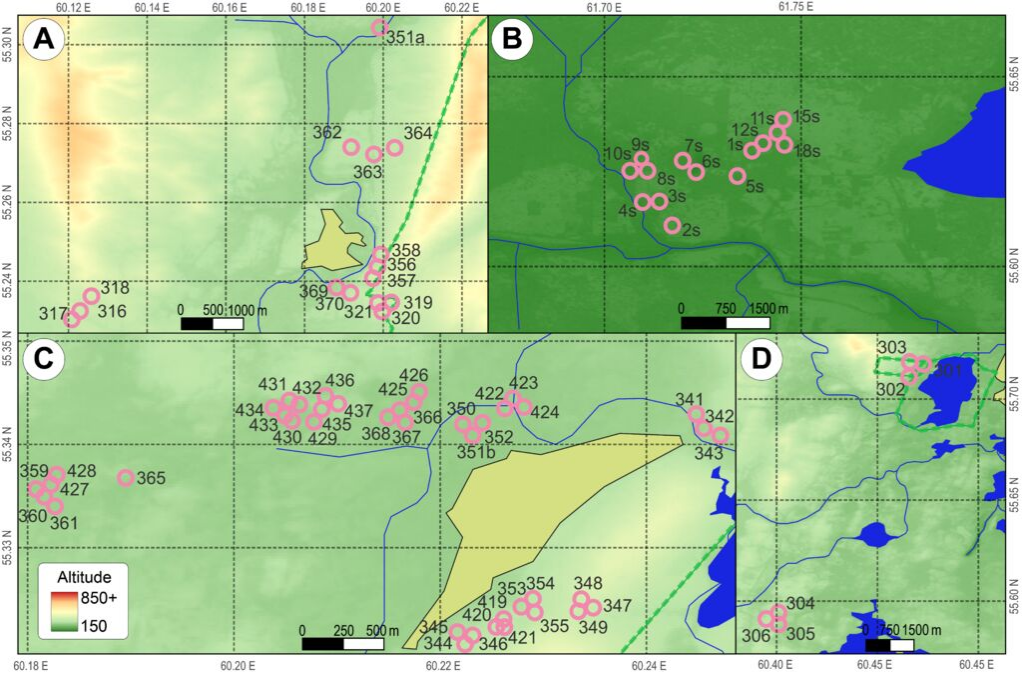
Maps of the sampling plots (local scale) near the following localities: **A** – Novotagilka, **B** – Sultanovskoye, **С** – Novoandreevka, **D** – Sugomak.

Most of the research was conducted in the vicinity of the Karabash copper smelter (KaCS), located 90 km northwest of Chelyabinsk (the Southern Urals) and in operation since 1910. The KaCS is one of Russia’s largest point sources of environmental pollution by heavy metals and sulphur dioxide. An industrial wasteland has arisen in its immediate surrounding area; this is a barren "moonscape" almost devoid of vegetation. According to our data, habitat quality becomes satisfactory for most insectivores and rodents at a distance of 9–11 km from the source of emissions (Mukhacheva et al. 2010a, Mukhacheva et al. 2010b). Background test plots (n = 62, Table 1) were located in four separate districts of the Chelyabinsk Oblast (Kyshtymsky, Karabashsky, Argayashsky and Miassky) at different distances (from 18 to 32 km) from the source of emissions (Figs 1, 2). Additional information on the occurrence of small mammals in various types of background (unpolluted) habitats (18 test plots) was obtained in 2007 during a single environmental examination of the Sultanovskoye copper-pyrite ore deposit before its development (near the village of Muslyumovo, Kunashaksky District, Chelyabinsk Oblast). At present, this area is a technogenic landscape, formed by a system of quarries.

According to geobotanical zoning, all surveyed areas are located in the pine-birch forests subzone on the eastern slopes of the Urals (Kulikov 2005). A total of 14 main habitat types – differing in terms of terrain and vegetation – were surveyed; these encompassed forests, sparse birch stand, meadow, swamp, borderland, a household waste dump, ruderal vegetation and a temporary camp.

The identification of different variants of habitats is based on a preliminary geobotanical survey of the background areas. For example, the studies of 2012–2014 used 42 variables to produce a comparative description of the test plots (n = 19); these comparised seven variables for characterising landscape and climatic conditions, 25 – for vegetation and soil and 10 – for assessing the degree of toxic pollution of territories (Table 2).

### Design description

The dataset includes the occurrence of species of small insectivores (Eulipotyphla) and rodents (Rodentia) within five administrative districts of the Chelyabinsk Oblast (Southern Urals, Russia). The collection of animals was carried out for eight years (2007–2014) during the snowless periods: this being either June–July (2007, 2011–2014) or September-October (2008–2010). In total, there were 62 observed test plots, covering a total of 14 main habitat types: forests (pine, birch, mixed and floodplain), sparse birch stand, meadows (marshy, dry), swamps (reed, bogged), border areas (pine forest-reed swamp, birch forest-dry meadow), a household waste dump, ruderal vegetation and a temporary camp.

### Funding

The research was supported by the Ecological Monitoring Program (no. 2007-10008), the Russian Foundation for Basic Research RFBR (projects no. 08-04-91766, no. 12-05-00811).

## Sampling methods

### Study extent

The dataset (Mukhacheva et al. 2021) is based on the records from the field logs. The coordinate reference to each mammal occurrence is given for the first time in the dataset. The majority of the dataset was obtained in the vicinity of the Karabash copper smelter (KaCS) during 2008–2014 (Mukhacheva et al. 2010a, Mukhacheva et al. 2010b, Mukhacheva et al. 2012, Mukhacheva and Davydova 2016). Moreover, some data were obtained in 2007 during a single environmental examination of the Sultanovskoye copper-pyrite ore deposit before its development (Kunashaksky District, Chelyabinsk Oblast) (Mukhacheva et al. 2009).

### Sampling description

Sampling was designed to cover the main habitat types for small mammals. The animals were caught using wooden traps (snap or live traps) arranged in lines (each line consisting of 10 to 25 traps) at a distance of 7–10 m from each other and exposed for 2–4 days, inspected once a day. Pieces of black bread with unrefined sunflower oil were used as bait for snap-traps. Live traps were baited with carrot, apple, oats and grass or moss to provide food and thermal comfort for captured specimens. In the period 2012–2014, modified lines were used to capture animals, consisting of alternating snap traps and live traps (in a ratio of 3:1). Thus, it was possible to keep records of animals in small-sized areas (which fit into the selected habitat options with highly mosaic environmental conditions), while, at the same time, catching species that "prefer" different trapping methods.

All collected animals were examined to determine their sex, age and reproductive status. In addition, the main exterior features (body weight, body, tail and foot length) and interior features (liver, kidney, heart, stomach and reproductive organs mass) were also evaluated. The identification by species of the sampled animals was carried out in the laboratory for ecotoxicology of populations and communities of the Institute of Plant and Animal Ecology, RAS. Latin species names and their order of mention in Table 3 are in accordance with Mammal Species of the World (Wilson and Reeder 2005).

Tissue samples were taken from most individuals for genetic and chemical analysis (in order to determine the concentrations of heavy metals in the liver, kidneys, skeleton and stomach contents). In addition, organ and tissue samples (from the liver, kidneys, testes) were taken from the most widespread "model" species (*Apodemusuralensis, Myodesglareolus, Myodesrutilus, Microtusarvalis, Microtusagrestis*) for histological analysis.

All applicable international, national and institutional guidelines for the care and use of animals were followed. This research was approved by the local ethics committee of the IPAE RAS.

### Quality control

Collected materials (skulls and samples of organs) are stored at the Institute of Plant and Animal Ecology (IPAE UB RAS, Yekaterinburg). All captured animals were determined to species level by qualified technicians using regional field guides (Gromov and Erbaeva 1995, Zaitsev et al. 2014).

## Geographic coverage

### Description

The data were collected on five administrative districts (Kyshtymsky, Karabashsky, Kunashaksky, Argayashsky and Miassky) of the Chelyabinsk Oblast Russia. All background areas were located in the central mountains, in the pine-birch forest subzone (Kulikov 2005). Most of the test plots (n = 62) were located along the macroslope of the South Urals, extending for almost 80 km from north to south. Geographical position, orography and soil and vegetation types all had an influence on the high faunistic richness of this site.

The geographical references were carried out by fixing the coordinates of the meeting point of the animals using a GPS Navigator (eTrex Legend Cх, Garmin, USA); the measurement error of the coordinates ranged from 10 to 70 m. In all records, the WGS-84 coordinate system was used.

### Coordinates

55.231 and 55.718 Latitude; 60.124 and 61.764 Longitude.

## Taxonomic coverage

### Description

Our dataset contains records of 18 species of small mammals, including five insectivorous species (Eulipotyphla) of one family (Soricidae) and 13 rodent species (Rodentia) of four families (Sciuridae, Dipodidae, Muridae and Cricetidae). We identified all the mammals to the species level, with the exception of the common vole (*Microtusarvalis*) and the East European vole (*M.levis*). These two twin species of rodents occur sympatrically on the territory of the Chelyabinsk Oblast, but cannot be separated morphologically. Genetic studies of these species have not been conducted; therefore, the records of the occurrence of *M.arvalis* include those of *M.levis*. The taxonomic identification of animals (to the species level) was determined according to specialised guidelines (Gromov and Erbaeva 1995, Zaitsev et al. 2014) and is included in this database according to GBIF.

The family Cricetidae accounted for both the highest number of species represented in the dataset (seven species, 39%) and the largest fraction of individual specimens in the generalised sample (more than 50%, 359 individuals). The second-highest number of species (five species, 28% of the total) and proportion of specimens (28%, 195 individuals) came from the Soricidae family of small insectivores. The third place in this list is occupied by representatives of the family Muridae, with four species (22% of the species list) and 152 individuals (21% of the total). The list is completed by representatives of the families Sciuridae and Dipodidae, with one species of each being found sporadically in the surveyed territories.

The distribution of species of small mammals in different habitats in the background areas was representative of the landscape and ecological state of the study territory and animal communities as a whole. The occurrence of different species in the studied variants of habitats is shown in Table 3.

Amongst the studied habitats, the largest number of species was recorded in birch forest (12 species), followed by floodplain forests and reed swamps (each with 11 species) and the household waste dump (nine species). By contrast, the smallest number of species was recorded in areas with ruderal vegetation (0) and in the temporary camp area and border zones (one each). The probable reason for this was the short catching period (comprising one recording session on the first test plot in each habitat variant).

The herb field mouse (*Apodemusuralensis*) is a prime example of a generalist species. It was recorded in most variants (10 out of 14) of the studied habitats, being found in forests (mainly birch), open habitats and the temporary camp. A large group of species – representatives of the genera Sorex, Myodes and Microtus – were found in 5–7 habitat variants (Table 3). At the same time, some species (*Sorexisodon*, *Neomysfodiens*, *Tamiassibiricus*, *Sicistabetulina*, *Micromysminutus*, *Apodemusagrarius*), due to their stenotopic and/or low abundance in this area, were recorded only in one of the habitats.

### Taxa included

**Table:** 

Rank	Scientific Name	Common Name
kingdom	Animalia	Animals
class	Mammalia	Mammals
order	Eulipotyphla	Insectivores
family	Soricidae	Shrews
order	Rodentia	Rodents
family	Sciuridae	Squirrels
family	Dipodidae	Dipodids
family	Muridae	Murids
family	Cricetidae	Hamsters

## Temporal coverage

### Notes

2007-06-23 through to 2014-07-17

## Usage licence

### Usage licence

Other

### IP rights notes

This work is licensed under a Creative Commons Attribution (CC-BY) 4.0 License.

## Data resources

### Data package title

Small mammals of background areas in the vicinity of the Karabash copper smelter (Southern Urals, Russia)

### Resource link

https://www.gbif.org/dataset/fe127afe-2458-4e9a-8b06-f7f4730d4103

### Alternative identifiers

http://gbif.ru:8080/ipt/resource?r=small_mammals_2021

### Number of data sets

1

### Data set 1.

#### Data set name

Small mammals of background areas in the vicinity of the Karabash copper smelter (Southern Urals, Russia)

#### Data format

Darwin Core

#### Number of columns

63

#### Description

The dataset contains records of small mammals (Eulipotyphla and Rodentia) collected on the background areas in the vicinity of the Karabash copper smelter (Southern Urals, Russia) and the territory of the Sultanovskoye deposit of copper-pyrite ores before the start of its development (Mukhacheva et al. 2021). Data were collected during the snowless periods in 2007 (18 sampling plots annually), 2008–2010 (13 plots annually), 2011 (30 plots) and 2012–2014 (19 plots annually). The capture of animals was carried out in different types of forests (pine, birch, mixed and floodplain), sparse birch stands, swamp (reed, bogged), marshy and dry meadows, border areas, a household waste dump, areas of ruderal vegetation and a temporary camp. Our studies of small mammals were conducted by trap lines (snap and live traps). During the study period, 709 specimens of small mammals were caught, which belong to five species of shrews and 13 species of rodents. The dataset may be highly useful for studying regional fauna and the distribution of species in different habitats and could also be used as reference values for environmental monitoring and conservation activities. We have published several faunal and analytical works, based on the materials collected in 2007 (Mukhacheva et al. 2009), in 2008–2010 (Mukhacheva et al. 2010a, Mukhacheva et al. 2010b, Mukhacheva and Davydova 2012), in 2011 (Mukhacheva et al. 2012); and 2012–2014 (Mukhacheva and Davydova 2016).

**Data set 1.** 

Column label	Column description
occurrenceID	An identifier for the Occurrence (as opposed to a particular digital record of the occurrence). In the absence of a persistent global unique identifier, construct one from a combination of identifiers in the record that will most closely make the occurrenceID globally unique. A variable.
type	The nature or genre of the resource. A variable.
modified	The most recent date-time on which the resource was changed. A constant ("DD-MM-YYYY").
language	A language of the resource. A constant ("en" = English).
licence	A legal document giving official permission to do something with the resource. A constant ("CC_BY_4_0" = Creative Commons Attribution (CC-BY) 4.0 Licence).
bibliographicCitation	A bibliographic reference for the resource as a statement indicating how this record should be cited (attributed) when used. A variable.
references	A related resource that is referenced, cited or otherwise pointed to by the described resource. A variable.
institutionCode	The name (or acronym) in use by the institution having custody of the object(s) or information referred to in the record. A constant ("Institute of Plant and Animal Ecology (IPAE), UB RAS").
datasetName	The name identifying the dataset from which the record was derived. A constant ("Small mammals of background areas in the vicinity of the Karabash copper smelter (Southern Urals, Russia)").
basisOfRecord	The specific nature of the data record. A variable.
catalogNumber	An identifier (preferably unique) for the record within the dataset or collection. A variable.
recordNumber	An identifier given to the Occurrence at the time it was recorded. Often serves as a link between field notes and an Occurrence record, such as a specimen collector's number. A variable, constructed by sample plot name ("L.") and catalogue number ("No.").
recordedBy	A list (concatenated and separated) of names of people, groups or organisations responsible for recording the original Occurrence. The primary collector or observer, especially one who applies a personal identifier (recordNumber), should be listed first. A constant ("Mukhacheva S.V. | Davydova Yu.A").
individualCount	The number of individuals present at the time of the Occurrence. A constant ("1").
sex	The sex of the biological individual(s) represented in the Occurrence. A variable.
lifeStage	The age class or life stage of the Organism(s) at the time the Occurrence was recorded. A variable.
occurrenceStatus	A statement about the presence or absence of a Taxon at a Location. A variable.
preparations	A list (concatenated and separated) of preparations and preservation methods for a specimen. A variable.
disposition	The current state of a specimen with respect to the collection identified in collectionCode or collectionID. A variable.
occurrenceRemarks	Comments or notes about the Occurrence. A variable.
identifiedBy	A list (concatenated and separated) of names of people, groups or organisations who assigned the Taxon to the subject. A variable.
dateIdentified	The date on which the subject was determined as representing the Taxon. A variable.
identificationReferences	A list (concatenated and separated) of references (publication, global unique identifier, URI) used in the Identification. A constant ("Gromov, Erbaeva 1995 | Zaitsev et al. 2014").
identificationRemarks	Comments or notes about the Identification. A variable.
scientificName	The full scientific name, with authorship and date information, if known. When forming part of an Identification, this should be the name in the lowest level taxonomic rank that can be determined. This term should not contain identification qualifications, which should instead be supplied in the IdentificationQualifier term. A variable.
acceptedNameUsage	The full name, with authorship and date information, if known, of the currently valid (zoological) or accepted (botanical) taxon. A variable.
kingdom	The full scientific name of the kingdom in which the taxon is classified. A constant ("Animalia").
phylum	The full scientific name of the phylum or division in which the taxon is classified. A constant ("Chordata").
class	The full scientific name of the class in which the taxon is classified. A constant ("Mammalia").
order	The full scientific name of the order in which the taxon is classified. A variable.
family	The full scientific name of the family in which the taxon is classified. A variable.
genus	The full scientific name of the genus in which the taxon is classified. A variable.
specificEpithet	The name of the first or species epithet of the scientificName. A variable.
taxonRank	The taxonomic rank of the most specific name in the scientificName. A constant ("SPECIES").
scientificNameAuthorship	The authorship information for the scientificName, formatted according to the conventions of the applicable nomenclaturalCode. A variable.
parentEventID	An identifier for the broader Event that groups this and potentially other Events. A variable.
eventID	An identifier for the set of information associated with an Event (something that occurs at a place and time). May be a global unique identifier or an identifier specific to the dataset. A variable, constructed by sample plot name ("l.") and event date ("DD-MM-YYYY").
fieldNumber	An identifier given to the event in the field. Often serves as a link between field notes and the Event. A variable.
eventDate	The date-time or interval during which an Event occurred. For occurrences, this is the date-time when the event was recorded. Not suitable for a time in a geological context. A variable ("DD-MM-YYYY").
year	The four-digit year in which the Event occurred, according to the Common Era Calendar. A variable.
month	The integer month in which the Event occurred. A variable.
day	The integer day of the month on which the Event occurred. A variable.
habitat	A category or description of the habitat in which the Event occurred. A variable.
samplingProtocol	The names of, references to, or descriptions of the methods or protocols used during an Event. A variable.
sampleSizeValue	A numeric value for a measurement of the size (time duration, length, area or volume) of a sample in a sampling event. A variable.
sampleSizeUnit	The unit of measurement of the size (time duration, length, area or volume) of a sample in a sampling event. A variable.
samplingEffort	The amount of effort expended during an Event. A variable.
higherGeography	A list (concatenated and separated) of geographic names less specific than the information captured in the locality term. A constant ("Urals | South Ural").
continent	The name of the continent in which the Location occurs. A constant ("Europe | Asia").
country	The name of the country or major administrative unit in which the Location occurs. A constant ("Russia").
countryCode	The standard code for the country in which the Location occurs. A constant ("RU").
stateProvince	The name of the next smaller administrative region than country (state, province, canton, department, region etc.) in which the Location occurs. A constant ("Chelyabinsk").
county	The full, unabbreviated name of the next smaller administrative region than stateProvince (county, shire, department etc.) in which the Location occurs. A variable.
locality	The specific description of the place. A variable.
minimumElevationInMetres	The lower limit of the range of elevation (altitude, usually above sea level), in metres. A variable.
maximumElevationInMetres	The upper limit of the range of elevation (altitude, usually above sea level), in metres. A variable.
decimalLatitude	The geographic latitude (in decimal degrees, using the spatial reference system given in geodeticDatum) of the geographic centre of a Location. Positive values are north of the Equator, negative values are south of it. Legal values lie between -90 and 90, inclusive. A variable.
decimalLongitude	The geographic longitude (in decimal degrees, using the spatial reference system given in geodeticDatum) of the geographic centre of a Location. Positive values are east of the Greenwich Meridian, negative values are west of it. Legal values lie between -180 and 180, inclusive. A variable.
geodeticDatum	The ellipsoid, geodetic datum or spatial reference system (SRS) upon which the geographic coordinates given in decimalLatitude and decimalLongitude are based. A constant ("WGS84").
coordinateUncertaintyInMetres	The horizontal distance (in metres) from the given decimalLatitude and decimalLongitude describing the smallest circle containing the whole of the Location. Leave the value empty if the uncertainty is unknown, cannot be estimated or is not applicable (because there are no coordinates). Zero is not a valid value for this term. A variable.
georeferencedBy	A list (concatenated and separated) of names of people, groups or organisations who determined the georeference (spatial representation) for the Location. A constant ("Davydova Yu.A., Mukhacheva S.V.").
georeferencedDate	The date on which the Location was georeferenced. A constant ("27-08-2021").
rightsHolder	A person or organisation owning or managing rights over the resource. A constant ("Institute of Plant and Animal Ecology (IPAE), UB RAS").

## Additional information

Mukhacheva S, Davydova Yu, Sozontov A (2021). Small mammals of background areas in the vicinity of the Karabash copper smelter (Southern Urals, Russia). v.1.3. Institute of Plant and Animal Ecology (IPAE). Dataset/Samplingevent. http://gbif.ru:8080/ipt/resource?r=small_mammals_2021&v=1.3

## Figures and Tables

**Table 1.** Sampling plots.

**Sampling plot**	**Latitude**	**Longitude**	**County**	**Habitat**	**Sampling years**
1s	55.63148	61.74432	Kunashakskiy District	pine forest	2007
2s	55.61350	61.71573	Kunashakskiy District	birch forest	2007
3s	55.61736	61.71042	Kunashakskiy District	dry meadow	2007
4s	55.61721	61.70884	Kunashakskiy District	marshy meadow	2007
5s	55.62418	61.73577	Kunashakskiy District	dry meadow	2007
6s	55.62523	61.72468	Kunashakskiy District	birch forest	2007
7s	55.62683	61.72033	Kunashakskiy District	marshy meadow	2007
8s	55.62558	61.70976	Kunashakskiy District	birch forest	2007
9s	55.62596	61.70952	Kunashakskiy District	dry meadow	2007
10s	55.62556	61.70694	Kunashakskiy District	marshy meadow	2007
11s	55.63427	61.74694	Kunashakskiy District	reed swamp	2007
12s	55.63260	61.74485	Kunashakskiy District	forested bog	2007
13s	55.62936	61.74715	Kunashakskiy District	ruderal vegetation	2007
14s	55.63150	61.74605	Kunashakskiy District	swamp-pine forest border	2007
15s	55.63488	61.74713	Kunashakskiy District	birch-dry meadow border	2007
16s	55.62822	61.76285	Kunashakskiy District	birch forest	2007
17s	55.63005	61.76425	Kunashakskiy District	mixed forest (birch and pine)	2007
18s	55.63327	61.74768	Kunashakskiy District	shift camp	2007
301	55.71472	60.47073	Kyshtymskiy District	birch forest	2008, 2009, 2010
302	55.71302	60.46700	Kyshtymskiy District	birch forest	2008, 2009, 2010
303	55.71779	60.46803	Kyshtymskiy District	birch forest	2008, 2009, 2010
304	55.59158	60.40092	Karabashskiy District	birch forest	2008, 2009, 2010
305	55.59119	60.40091	Karabashskiy District	birch forest	2008, 2009, 2010
306	55.58982	60.40098	Karabashskiy District	birch forest	2008, 2009, 2010
316	55.23212	60.12398	Miasskiy District	birch forest	2008, 2009, 2010
317	55.23134	60.12361	Miasskiy District	birch forest	2008, 2009, 2010
318	55.23277	60.12412	Miasskiy District	birch forest	2008, 2009, 2010
319	55.23752	60.20392	Argayashskiy District	birch forest	2008, 2009, 2010
320	55.23627	60.20299	Argayashskiy District	birch forest	2008, 2009, 2010
321	55.23734	60.20294	Argayashskiy District	birch forest	2008, 2009, 2010
341	55.34162	60.24627	Karabashskiy District	floodplain forest	2011
342	55.34129	60.24649	Karabashskiy District	floodplain forest	2011
343	55.34112	60.24716	Karabashskiy District	floodplain forest	2011
344	55.32039	60.22241	Miasskiy District	mixed forest, slope	2011
345	55.32118	60.22233	Miasskiy District	mixed forest (birch and pine), slope	2011
346	55.32111	60.22355	Miasskiy District	mixed forest (birch and pine), slope	2011
347	55.32419	60.23483	Miasskiy District	mixed forest (birch and pine), top	2011
348	55.32476	60.23354	Miasskiy District	mixed forest (birch and pine), top	2011
349	55.32385	60.23339	Miasskiy District	mixed forest (birch and pine), top	2011
350	55.34194	60.22300	Miasskiy District	floodplain forest	2011
351a	55.30433	60.19909	Miasskiy District	floodplain forest	2010
351b	55.34179	60.22341	Miasskiy District	floodplain forest	2011
352	55.34209	60.22369	Miasskiy District	floodplain forest	2011
353	55.32389	60.22787	Miasskiy District	dry meadow	2011
354	55.3244	60.2286	Miasskiy District	dry meadow	2011
355	55.32387	60.22878	Miasskiy District	dry meadow	2011
356	55.24191	60.20079	Argayashskiy District	birch forest	2011
357	55.24111	60.19806	Argayashskiy District	birch forest	2011
358	55.24377	60.20291	Argayashskiy District	birch forest	2011
359	55.3354	60.18121	Miasskiy District	reed swamp	2011
360	55.33494	60.18161	Miasskiy District	reed swamp	2011
361	55.33452	60.18199	Miasskiy District	reed swamp	2011
362	55.27402	60.19184	Miasskiy District	pine forest	2011
363	55.27112	60.19796	Miasskiy District	pine forest	2011
364	55.27384	60.20291	Miasskiy District	pine forest	2011
365	55.33676	60.1895	Miasskiy District	floodplain forest	2011
366	55.34293	60.21609	Miasskiy District	dump of household waste	2011
367	55.34279	60.21607	Miasskiy District	dump of household waste	2011
368	55.34288	60.21574	Miasskiy District	dump of household waste	2011
369	55.23809	60.18916	Miasskiy District	dry meadow	2011
370	55.23739	60.19237	Miasskiy District	dry meadow	2011
419	55.32284	60.22602	Miasskiy District	birch forest	2012, 2013, 2014
420	55.32258	60.22582	Miasskiy District	birch forest	2012, 2013, 2014
421	55.32233	60.22544	Miasskiy District	birch forest	2012, 2013, 2014
422	55.34348	60.22613	Miasskiy District	floodplain forest	2012, 2013, 2014
423	55.34378	60.22665	Miasskiy District	floodplain forest	2012, 2013, 2014
424	55.34362	60.2272	Miasskiy District	floodplain forest	2012, 2013, 2014
425	55.34342	60.21677	Miasskiy District	dump of household waste	2012, 2013, 2014
426	55.34404	60.21731	Miasskiy District	dump of household waste	2012, 2013, 2014
427	55.33613	60.18226	Miasskiy District	reed swamp	2012, 2013, 2014
428	55.33680	60.18255	Miasskiy District	reed swamp	2012, 2013, 2014
429	55.34257	60.20763	Miasskiy District	marshy meadow	2012, 2013, 2014
430	55.34241	60.20563	Miasskiy District	marshy meadow	2012, 2013, 2014
431	55.34334	60.20609	Miasskiy District	marshy meadow	2012, 2013, 2014
432	55.34345	60.20563	Miasskiy District	sparse birch stand	2012, 2013, 2014
433	55.34280	60.20547	Miasskiy District	sparse birch stand	2012, 2013, 2014
434	55.34301	60.20486	Miasskiy District	sparse birch stand	2012, 2013, 2014
435	55.34341	60.20853	Miasskiy District	pine forest	2012, 2013, 2014
436	55.34376	60.20887	Miasskiy District	pine forest	2012, 2013, 2014
437	55.34353	60.20919	Miasskiy District	pine forest	2012, 2013, 2014

**Table 2.** Characteristics of the variables used to describe the parameters of the micro-environment in the vicinity of Karabash copper smelter (2012–2014).

**No**	**Variable**	**Units**	**Calculation procedure**
1	Height above sea level	m	Determination of indicators using a GPS navigator (eTrex Legend Cх, Garmin, USA) and a level (С410, Sokkia, Japan).
2	Slope ratio	degrees	
3	Average daily temperature in July	^°^С	Thermologgers (n = 61) Thermochron iButton DS1921G were installed on the soil surface (1–2 for each test plot) for 340 days (from July 2012 to June 2013). The readings were recorded 6 times a day (every 4 hours). Measurement range from –40°С to + 85°С, accuracy ± 0.5°C.
4	Minimal daily temperature in July	^°^С	
5	Maximum daily temperature in July	^°^С	
6	Daily temperature amplitude	^°^С	
7	Volume humidity of the horizons of A0+A1	%	Measurements with the HH2 Field Moisture Analyzer and ThetaProbe ML2 (Delta-T, UK). For all, checkpoints were performed in the same time frame (in dry weather).
8	Soil humidification	score	Phyto-indication analysis using ecological scales D.N. Tsyganov and the IBIS 6.1 programme.
9	Salt soil regime	score	
10	Rich soils with nitrogen	score	
11	Number of the wood tier species	sample	Based on full geobotanical descriptions of each test plot with a size of 625 m^2^ (25m × 25 m or 62.5 × 10 m)
12	Number of the shrub tier species	sample	
13	Number of the grass-bush tier species	sample	
14	Average diameter of trees	m	The arithmetic mean for all trees at three test plots of each habitat. The diameter of each tree (more than 0.05 m in diameter) was measured at chest level using a caliper with an accuracy of 0.01 m.
15	Average height of trees	m	The arithmetic mean for all trees at three test plots of each habitat. The height of each tree (more than 0.05 m in diameter) was calculated using the trunk diameter (in m) and the height equations.
16	Density of the woodland	sample/ha	The number of trees (with a diameter of more than 0.05 m) on three test plots per 1 ha.
17	Wood standing stock	m^3^/ha	The volume of wood, according to the data of a continuous enumeration for three test plots per 1 ha.
18	Stumps area	%	The test plot's relative total cross-sectional area of all stumps (more than 0.05 m in diameter). The diameter of the stump was measured at the base in two directions using a caliper.
19	Walleye area	%	The relative total cross-sectional area of trees that died within the test plots taking into account the degree of their decomposition and diameter.
20	Drywall area	%	
21	Projective coverage of the undergrowth	%	The relative total area occupied by this group, determined for each test plot, based on complete geobotanical descriptions.
22	Projective coverage of the shrub tier	%	
23	Projective coverage of the grass-bush tier	%	
24	Projective coverage of the mosses	%	
25	Projective coverage of horizon A0 or rags	%	The relative area of the projected forest litter or substratum, determined for each test plot.
26	Average height of the shrub tier	m	Arithmetic means based on 10 measurements (differentiated for each tier), determined for each test plot.
27	Average height of the grass-bush tier	m	
28	Average horizon power A0	m	Arithmetic means based on 20 measurements in 5–7 m, determined for each test plot.
29	Bare soil projective cover	%	The relative area devoid of vegetation and forest litter, determined for each test plot.
30	Projective stone coverage	%	Relative area occupied by stones, determined for each test plot.
31	Garbage	%	The relative area occupied by garbage, determined for each test plot
32	Illumination	%	Calculated based on photographs of the projection of the crowns of woody plants (n = 315) at the height of 40–50 cm from the soil surface at random points (7–10 test plots) with further image processing in the SIAMS Photolab package (v.4.0.4.x).
33	pH_water_ А0	unit рН	The measurements were carried out on a pH-410 potentiometer at a substrate/water ratio of 1:25 for forest litter and 1: 5 for mineral horizons.
34	pH_water_ А1	unit рН	
35 36 37 38 39 40 41 42	Concentration of Cu in А0 Concentration of Zn in А0 Concentration of Cd in А0 Concentration of Pb in А0 Concentration of Cu in А1 Concentration of Zn in А1 Concentration of Cd in А1 Concentration of Pb in А1	mkg/g	Mobile forms of heavy metals (Cu, Zn, Cd and Pb) were extracted from the samples with 5% nitric acid. The concentration of mobile forms of heavy metals (Cu, Zn, Cd and Pb) was determined by atomic absorption spectrometry on an ASS-6 Vario instrument (Analytik Jena, Germany).

**Table 3.** Occurrence (number of individuals) of the studied species in different types of habitats (1 – birch forest; 2 – pine forest; 3 – mixed forest; 4 – floodplain forest, 5 – sparse birch stand; 6 – dry meadow; 7 – marshy meadow; 8 – reed swamp; 9 – bogged swamp; 10 – birch-dry meadow border; 11 – swamp-pine forest border; 12 – household waste dump; 13 – temporary camp; 14 – ruderal vegetation. Latin species names and their order are given according to Mammals Species of the World (Wilson and Reeder 2005).

**Species**	**Type of habitats**														**Total** **individ.**
	**1**	**2**	**3**	**4**	**5**	**6**	**7**	**8**	**9**	**10**	**11**	**12**	**13**	**14**	
*Neomysfodiens* Pennant, 1771	0	0	0	0	0	0	0	2	0	0	0	0	0	0	2
*Sorexaraneus* Linnaeus, 1758	70	0	8	15	0	4	10	11	0	0	0	5	0	0	123
*Sorexcaecutiens* Laxmann, 1788	40	1	0	4	3	0	1	1	0	0	0	1	0	0	51
*Sorexisodon* Turov, 1924	1	0	0	0	0	0	0	0	0	0	0	0	0	0	1
*Sorexminutus* Linnaeus, 1766	1	0	0	9	2	0	3	2	0	0	0	1	0	0	18
*Tamiassibiricus* Laxmann, 1769	0	0	0	2	0	0	0	0	0	0	0	0	0	0	2
*Sicistabetulina* Pallas, 1779	1	0	0	0	0	0	0	0	0	0	0	0	0	0	1
*Arvicolaamphibius* Linnaeus, 1758	0	0	0	0	0	0	0	1	2	0	0	0	0	0	3
*Microtusagrestis* Linnaeus, 1761	5	0	2	9	10	0	16	2	0	0	0	0	0	0	44
*Microtusarvalis* Pallas, 1779	3	0	2	0	0	33	2	0	0	0	0	24	0	0	64
*Alexandromysoeconomus* Pallas, 1776	1	0	0	9	0	5	2	17	4	0	0	5	0	0	43
*Stenocraniusgregalis* Pallas, 1779	0	0	0	0	0	8	1	1	0	1	0	0	0	0	11
*Myodesglareolus* Schreber, 1780	84	4	2	76	3	0	0	3	0	0	0	7	0	0	179
*Myodesrutilus* Pallas, 1779	3	4	0	1	1	0	2	0	0	0	4	0	0	0	15
*Apodemusagrarius* Pallas, 1771	0	0	0	3	0	0	0	0	0	0	0	0	0	0	3
*Apodemusuralensis* Pallas, 1811	24	6	5	51	3	1	2	11	0	0	0	35	4	0	142
*Micromysminutus* Pallas, 1771	0	0	0	1	0	0	0	0	0	0	0	0	0	0	1
*Musmusculus* Linnaeus, 1758	1	1	0	0	0	0	1	2	0	0	0	1	0	0	6
